# Isolation and Characterization of Microorganisms from Buckwheat Farmland for the Bioconversion of Quercetin

**DOI:** 10.3390/microorganisms13061224

**Published:** 2025-05-27

**Authors:** Jiyoung Shin, Junho Yang, Beom-Su Cho, Jisoo Han, Ji-Young Yang

**Affiliations:** Department of Food Science and Technology, Pukyong National University, Busan 48513, Republic of Korea; nadia83@naver.com (J.S.); yjunho9@gmail.com (J.Y.); jbs4644@gmail.com (B.-S.C.);

**Keywords:** bioconversion, buckwheat, flavonoids, quercetin, isoquercetin, *Bacillus licheniformis*

## Abstract

The usability of flavonoids, which have numerous functional benefits, is limited by their low solubility. In this study, microorganisms were isolated from the soil of a buckwheat farmland located in Pyeongchang, Republic of Korea, to identify potential agents for flavonoid bioconversion. Strain 3P-1, which exhibited 98.9% 16S rRNA gene sequence similarity to *Bacillus licheniformis* strain IND706, demonstrated the ability to utilize flavonoids during fermentation. During the 7-day fermentation process with strain 3P-1, a significant decrease in quercetin content was observed, accompanied by the generation of an unknown compound. High-performance liquid chromatography coupled with mass spectrometry analysis of the unknown compound revealed its molecular weight. Among the four potential candidates identified, isoquercetin was determined to be the most likely agent for flavonoid bioconversion based on its biosynthetic pathway and substrate specificity, as well as known characteristics of strain 3P-1. These findings suggest that the isolated strain 3P-1 has substantial potential as a bioconversion agent for transforming quercetin to isoquercetin, which enhances its bioavailability.

## 1. Introduction

Flavonoids are a class of secondary plant phenolics that are widely distributed in nature and constitute a major part of the human diet. Flavonoids have a basic structure consisting of 15 carbon atoms (C_6_-C_3_-C_6_) and are categorized into several subclasses, including flavonols, flavones, flavanones, catechins, anthocyanidins, isoflavones, dihydroflavonols, and chalcones. They exhibit diverse biological properties, including antioxidant, free radical-scavenging, antitumor, and microcirculation-improving properties [1,2]. Quercetin is a prominent flavonoid found in various fruits, vegetables, grains, and seeds, and is well known for its antioxidant, anti-inflammatory, anticarcinogenic, and vasodilatory properties [3,4]. The structure of quercetin includes five hydroxyl (-OH) groups, which typically confer hydrophilic properties, but quercetin also possesses lipophilic characteristics, allowing for interactions with both water and lipid environments [5].

In the human diet, flavonoids are mostly present as O-glycosides bound to sugars such as glucose and rhamnose. Microbial fermentation plays a crucial role in flavonoid biotransformation. For instance, microbial β-glycosidase enzymes can break the O-β-glycosidic bonds in flavonoids, producing aglycones such as daidzein and genistein, which are readily absorbed and exhibit enhanced bioavailability [6]. Bacteria such as *Bifidobacterium pseudocatenulatum* and fungi such as *Aspergillus awamori* have been shown to produce aglycones from flavonoid glycosides, improving the compounds’ solubility and functionality [7].

Microbial bioconversion is a broad concept involving the transformation of organic compounds into structurally related compounds through enzymatic reactions [8]. This process is widely used in the food industry to manufacture various bioproducts, including enzymes, organic acids, and pigments, and is an eco-friendly method for converting waste to energy [8]. Overall, the interaction between flavonoids and microbes not only enhances the bioavailability and functional properties of these compounds but also plays a role in food processing and environmental sustainability. The composition and metabolic processes of microbes are influenced by environmental factors, which, in turn, affect the efficiency and outcomes of bioconversion processes [9]. Therefore, in this study, we aimed to evaluate the flavonoid bioconversion potential of strains isolated from a farm where flavonoid-rich buckwheat is cultivated. Analytical standards of quercetin, isoquercetin, and rutin were obtained from Sigma-Aldrich (St. Louis, MO, USA) and used for compound identification and comparison.

## 2. Materials and Methods

### 2.1. Soil Collection and Bacterial Isolation

Soil samples were collected from a buckwheat farm in Pyeongchang, Kangwon-do, Republic of Korea. The collected soil samples were stored at 4 °C in a refrigerator until further use. For bacterial isolation, 2 g of the soil from each sample was resuspended in phosphate-buffered saline (PBS). Aliquots of the diluted (10^−1^ and 10^−2^) suspensions were inoculated on selective media, including tryptic soy agar (TSA, Becton Dickinson and Co., Sparks, MD) and Reasoner’s 2A agar (R2A, Becton Dickinson and Co.). The inoculated plates were incubated at 30 °C for 3 days. Following incubation, morphologically distinct bacterial colonies were observed, and strains were selected for further analysis.

### 2.2. Bacterial Identification

**Figure 1:**
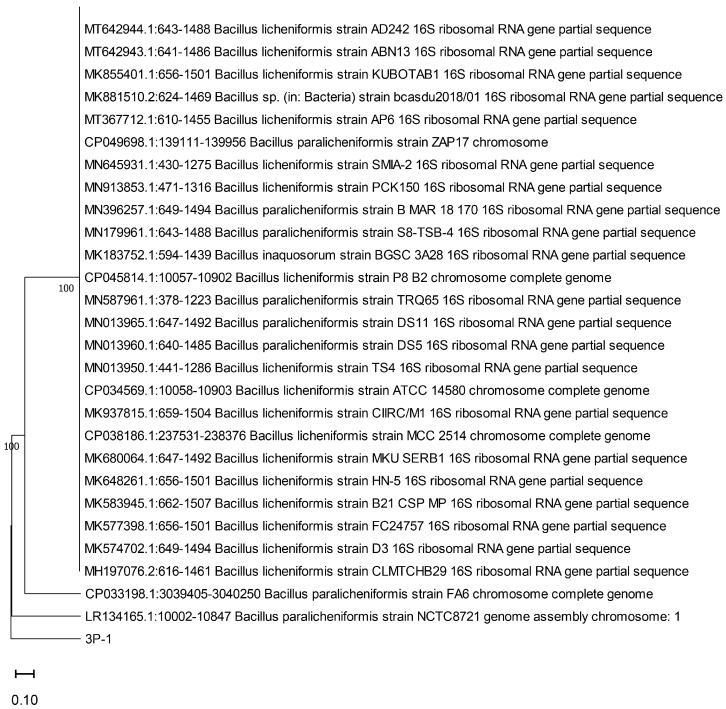
Phylogenetic tree based on the 16S rRNA gene sequence of strain 3P-1 and related bacterial strains. The evolutionary history was inferred using the neighbor-joining method. The optimal tree is shown, and the percentage of replicate trees in which the associated taxa clustered together in the bootstrap test (500 replicates) is indicated at the nodes. The tree is drawn to scale, with branch lengths representing the number of base differences per site. Evolutionary distances were computed using the p-distance method. Bootstrap values were generated using a random resampling method.

Bacteria were identified using 16S rRNA gene analysis following the method described by Ponnusamy et al. [10]. Genomic DNA was extracted from the isolated strains using the Accuprep^®^ Genomic DNA Extraction Kit (Bioneer, Daejeon, Republic of Korea) according to the manufacturer’s instructions. 3P-F (TAAACGATGAGTGCTAAGT), which was newly designed to prevent frameshift, and the universal primer 1492R (GGTTACCTTGTTACGACTT) were utilized. The polymerase chain reaction (PCR) mixture contained 2 μL of template DNA, 1 μL of each primer at 0.2 μmol concentrations, and 10 μL of Prime Taq Premix (G-3000; GeNet Bio, Daejeon, Republic of Korea). PCR was conducted using AllInOneCycler™ (Bioneer, Daejeon, Republic of Korea) with the following conditions: initial denaturation at 94 °C for 5 min, followed by 35 cycles at 94 °C for 30 s, 50 °C for 30 s, and 72 °C for 30 s, with a final extension at 72 °C for 10 min. The PCR products were electrophoresed on 1.5% agarose gel stained with EcoDye Nucleic Acid Staining Solution (Biofact, Daejeon, Republic of Korea) to confirm successful amplification. The amplified products were purified using a Bioneer purification kit, according to the manufacturer’s instructions. Purified 16S rRNA gene fragments were sequenced by Bioneer and analyzed using BioEdit software (https://bioedit.software.informer.com/, accessed date: 1 November 2021) for alignment. A phylogenetic tree was constructed using the neighbor-joining method in MEGA X software with 500 bootstrap values [11]. The evolutionary history was inferred using the neighbor-joining method [12]. The phylogenetic tree was constructed using the MEGA X software (version 11) [13]. Multiple sequence alignments were performed with the built-in MUSCLE algorithm using default parameters. The evolutionary history was inferred using the neighbor-joining (NJ) method, and evolutionary distances were computed using the p-distance method, which measures the proportion of nucleotide sites at which the sequences differ [14]. All sequences were trimmed to 846 bp to ensure uniformity before alignment. Sequences of closely related strains were retrieved from the NCBI GenBank database for performing a comparative analysis. An unrooted phylogenetic tree was constructed without assigning an explicit outgroup. The robustness of the inferred tree was evaluated through bootstrap analysis with 500 replicates. The percentage of replicate trees in which the associated taxa clustered together is indicated next to the branches of the phylogenetic tree. The tree was drawn to scale, with branch lengths representing the number of base differences per site, consistent with the evolutionary distances used. All ambiguous positions were removed on a pairwise basis using the pairwise deletion option. The percentage of replicate trees in which the associated taxa clustered together in the bootstrap test (500 replicates) is shown below the branches in Figure 1 [13]. The tree is drawn to scale, with branch lengths in the same units as those of the evolutionary distances used to infer the phylogenetic tree. The evolutionary distances were computed using the p-distance method [14]; they are presented as units of the number of base differences per site.

### 2.3. Screening of Flavonoid-Converting Bacteria

The isolated strains were then examined for their flavonoid-bioconversion potential. Each strain was cultured in tryptic soy broth containing 200 mg/L rutin and quercetin, flavonoids typically present in buckwheat, at 35 °C. Samples were collected every day for 7 days. The samples were stored in a freezer at −80 °C and used to analyze the content of rutin and quercetin.

### 2.4. Rutin and Quercetin Quantification Using HPLC

The flavonoid content was determined using HPLC on a U-3000 device (Thermo Fisher Scientific Inc., Waltham, MA, USA). Before analysis, all samples were passed through a 0.45 μm cellulose acetate syringe filter (Adventec, Tokyo, Japan) to remove any particulates. Chromatographic separation was achieved using an Acclaim C18 column (250 mm × 4.6 mm, 5 μm; Thermo Fisher Scientific Inc.). The mobile phases employed were 0.03 M phosphoric acid (A) and methanol (B). The elution gradient was as follows: 0 min, 60% A, 40% B; 10 min, 0% A, 100% B; 15 min, 0% A, 100% B; 20 min, 60% A, 40% B; and 25 min, 60% A, 40% B. The flow rate was maintained at 1.0 mL/min and the detection wavelength was set at 360 nm. Each sample injection volume was 20 μL. A calibration curve was established under these conditions to enable simultaneous quantification of rutin and quercetin. In order to verify the identity of compounds generated during the bioconversion process, a comparative analysis was carried out using commercially available reference standards, analyzed under identical chromatographic conditions.

### 2.5. Unknown Compound Analysis Using HPLC-TOF/MS

HPLC/MS was performed to determine the molecular weights of unknow compound. Samples were analyzed using liquid chromatography/quantitative time-of-flight mass spectrometry (LC/Q-TOF)/MS. HPLC analysis was performed using an Acquity I-Class device (Waters Corporation, Milford, MA, USA). The Q-TOF MS analysis was performed using a Maxis HD device (Bruker, Karlsruhe, Germany). The column temperature was 40 °C, and 0.1% formic acid in water (A) and 0.1% formic acid in acetonitrile (B) were used. The elution gradient was as follows: 0 min, 99% A, 1% B; 2 min, 99% A, 1% B; 12 min, 20% A, 80% B; 16 min, 0% A, 100% B; 18 min, 0% A, 100% B; 19 min, 99% A, 1% B; and 21 min, 99% A, 1% B. The flow rate was 0.4 mL/min.

## 3. Results and Discussion

### 3.1. Isolation of Bacteria

Molecular identification using 16S rRNA gene sequence analysis provided information on strains with high genetic similarity to other strains. The 3P-1 strain displayed 98.9% similarity to *Bacillus licheniformis* strain IND 706, which is a Gram-positive bacterium commonly found in soil. This analysis involved 30 nucleotide sequences. All ambiguous positions were removed for each sequence pair (pairwise deletion option). There were 956 positions in the final dataset. Evolutionary analyses were conducted in MEGA11 [11]. The neighbor-joining method was used to construct four phylogenetic trees by comparing the sequences of the novel strains with those from the NCBI database.

### 3.2. Quercetin Bioconversion

**Figure 2:**
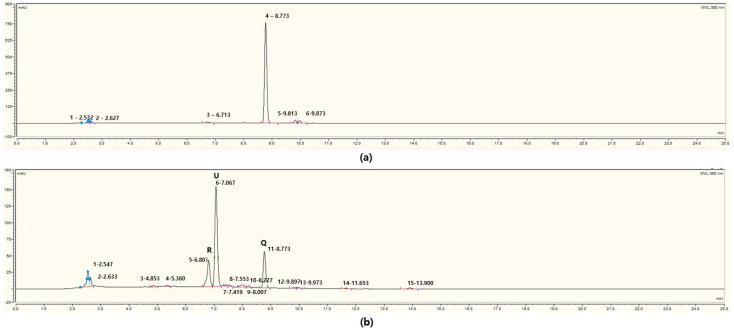
Comparison of high-performance liquid chromatography (HPLC) chromatograms (**a**) before and (**b**) after quercetin bioconversion. Peaks were detected at 360 nm. The retention times (RTs) of major compounds are as follows: rutin (R), RT = 6.807 min; unknown compound (U), RT = 7.067 min; quercetin (Q), RT = 8.773 min.

Quercetin was bioconverted by strain 3P-1, producing an unidentified compound. The HPLC analysis (Figure 2) revealed the quercetin peak before fermentation (Figure 2a). After 7 days of fermentation, three prominent peaks were observed: quercetin at a retention time of 8.77 min, rutin at 6.8 min, and a third peak, slightly shifted by 0.26 min from the rutin peak, representing the unidentified compound (Figure 2b).

### 3.3. Changes in Flavonoid Content Before and After Bioconversion Using 3P-1

**Figure 3:**
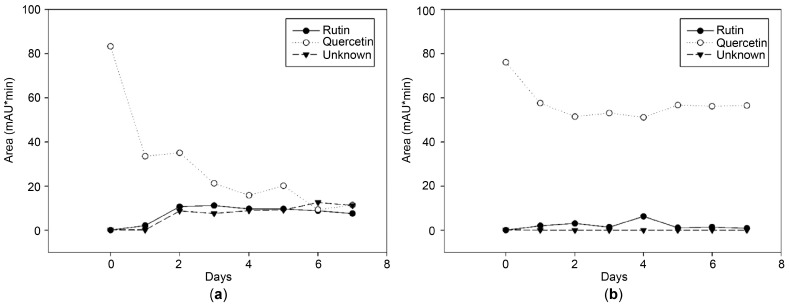
Changes in flavonoid composition in tryptic soy broth (TSB) medium containing 200 ppm quercetin over 7 days: (**a**) fermented with strain 3P-1; (**b**) unfermented control without strain 3P-1. Rutin, quercetin, and an unknown compound were quantified based on peak area (mAU·min).

Figure 3 illustrates the changes in flavonoid content in the presence or absence of strain 3P-1 and 200 mg/L quercetin. In the control group without any microbial inoculation (Figure 3b), the degradation of quercetin was presumed to have occurred due to chemical oxidation [15,16]. However, in the treatment sample supplemented with the isolated strain, 3P-1 (Figure 3a), the relative content of quercetin decreased to a greater extent than that in the control. This reduction was accompanied by an increase in the relative levels of isoquercetin and rutin, suggesting microbial involvement in the glycosylation of quercetin. On day 1, the content of quercetin, rutin, and the unknown compound was 83.28, 0, and 0 mAU·min, and on day 7, it was 11.45, 7.60, and 11.28 mAU·min, respectively. Furthermore, the rutin content increased until day 4 and then decreased. In contrast, no changes in flavonoid content were observed in media containing 3P-1 (Figure 3b). In both the control group and the group inoculated with the isolated strain, the quercetin content significantly decreased on day 1. The reduction rate in the inoculated group was more than twice that of the control group, with a corresponding increase in the levels of rutin and the unknown compound. A decreasing trend in quercetin content continued on day 2. Although the levels of rutin and the unknown compound did not increase after day 2 in the inoculated group, quercetin degradation persisted. These results indicate that the bacterial strain uses and degrades quercetin during the incubation period. It should be noted that the quantification method used in this study was based on peak area, which represents a relative, rather than absolute, measurement; therefore, standard compounds were not used for absolute quantification. Although this approach does not allow for precise determination of reaction yields, it enables the comparison of relative changes in compound abundance. The observed conversion of quercetin to the unknown compound and subsequently to rutin in the presence of the strain is presumed to be mediated by enzymatic activity. The unknown compound was not quantified using a standard substance; therefore, the flavonoid content is expressed as peak area (mAU·min).

*Bacillus* spp. ferment flavonoids and phenolic compounds [17]. For example, *Bacillus subtilis* produces daidzein and genistein in *cheonggukjang* (fermented soybean paste), and *B. pumilus* HY1 increases isoflavone aglycone, flavonol, and gallic acid concentrations [18]. Additionally, *B. subtilis* fermentation of soybeans yields chlorogenic acid and naringenin [19]. *Bacillus cereus* converts quercetin to isoquercetin during fermentation, with a yield of 20% [20]. Bacteria, except for some *Bacillus* species, can convert quercetin to other forms of flavonoids and phenolic compounds [16].

### 3.4. HPLC/MS Analysis of Fermented Flavonoids Using Isolated Bacteria

**Figure 4:**
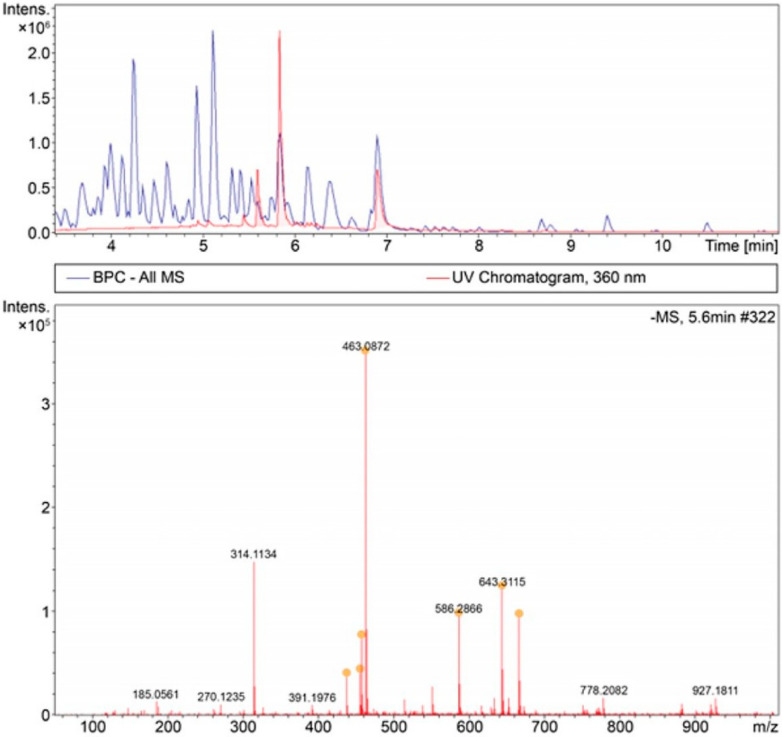
High-performance liquid chromatography/mass spectrometry (HPLC/MS) analysis to identify the unknown peak observed in the HPLC analysis. Orange dots represent the major precursor ions selected for MS/MS fragmentation in the LC/Q-TOF-MS analysis.

The fermentation products were analyzed using HPLC, and HPLC/MS was performed to determine the molecular weight of the unidentified compound. During the HPLC analysis, a novel peak was observed and separated into two distinct peaks at retention times of 5.6 and 5.8 min, in addition to rutin at 5.4 min and quercetin at 6.9 min (Figure 4).

The spectrum analysis revealed that the peak at 5.6 min corresponded to molecular weights of 437.2754, 455.2130, 463.0872, and 586.2866 g/mol. The peak at 5.8 min corresponded to molecular weights of 463.0872 and 563.1039 g/mol. The chemical ion formulae were determined based on the molecular weights, as listed in Table 1. Compounds corresponding to these formulae were identified using a PubChem search.

The compound with the molecular weight of 463.0872 g/mol was matched to four possible substances: myricetin 3-O-α-L-rhamnoside, quercetin-3-O-galactoside (hyperoside), isoquercetin (quercetin-3-glucoside), and delphinidin-3-O-glucoside. Based on the relationship between the biosynthetic pathway and the precursor compounds, isoquercetin was identified as the most probable substance. Myricetin 3-O-α-L-rhamnoside is synthesized from myricetin and UDP-β-L-rhamnose, quercetin-3-O-galactoside is synthesized from quercetin and UDP-α-D-galactose through the action of quercetin-O-glucosyltransferase, and delphinidin-3-O-glucoside is synthesized using UDP-α-D-glucose and delphinidin through the action of anthocyanidin 3-O-glucosyltransferase in the anthocyanin biosynthesis pathway. Isoquercetin is synthesized from not only quercetin and α-D-glucose by the action of quercetin-3-O-glucosyltransferase but also from rutin degradation. Given the experimental conditions with the media containing glucose and quercetin and the observed changes in flavonoid content, isoquercetin was identified as the most likely compound produced. Isoquercetin is a glycosylated form of quercetin in which a glucose molecule is attached to the hydroxyl group of quercetin. This glycosylation increases the water solubility of isoquercetin, enhancing its bioavailability compared with that of quercetin, enabling it to be more easily absorbed in the gastrointestinal tract and potentially exert greater biological benefits [21,22].

Isoquercetin, a monoglucoside derivative of quercetin, has substantially better physicochemical and pharmacokinetic properties than its aglycone. In particular, isoquercetin demonstrates approximately 20-fold greater intestinal absorption and has been classified as generally recognized as safe (GRAS) by the United States Food and Drug Administration (FDA) [23]. This enhanced bioavailability is attributed to its increased water solubility conferred by glycosylation. Isoquercetin is enzymatically produced via glucosylation at the 3-hydroxyl group of quercetin by quercetin-3-O-glucosyltransferase and can subsequently be converted to rutin through the action of rutin synthase and rhamnosyltransferase [24]. Notably, during fermentation, *B. cereus* converts quercetin to isoquercetin with a yield of 20% but does not convert rutin to isoquercetin [20]. These findings are consistent with the results of our study, confirming that isoquercetin is synthesized through glucosylation of quercetin.

Isoflavonoids are plant secondary metabolites biosynthesized from the 2-phenylchroman backbone of flavonoids, and they are particularly abundant in the Papilionoideae subfamily of the Leguminosae family. Similarly, isoquercetin is also a plant-derived secondary metabolite, formed from quercetin, and is commonly found in nature. Among various structural modifications, glycosylation plays a key role in enhancing and diversifying the biological functions of natural products and secondary metabolites. This process is catalyzed by UDP-glycosyltransferases, which form glycosidic bonds by transferring a sugar moiety from a donor molecule to an acceptor [25].

**Figure 5:**
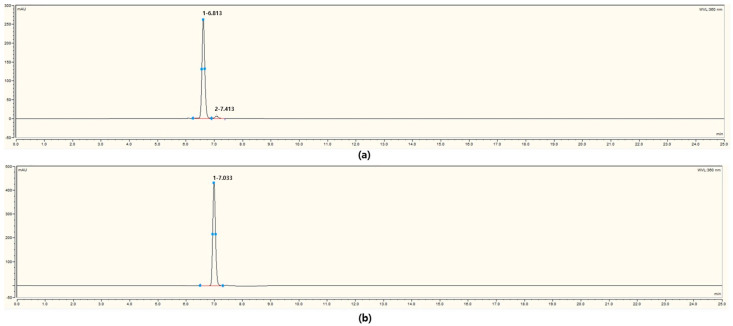
High-performance liquid chromatography (HPLC) chromatograms of flavonoid standards used for peak identification. (**a**) Rutin at 100 ppm with a retention time of 6.813 min and peak area of 39.1909 mAU·min; (**b**) isoquercetin (quercetin-3-O-glucoside) with a retention time of 7.033 min and peak area of 48.4411 mAU·min.

Under the same HPLC analytical conditions, standard compounds were analyzed as shown in Figure 5, and peaks corresponding to rutin and isoquercetin were identified as shown in Figure 3. At a concentration of 100 ppm, partial overlap of the peaks was expected owing to the proximity of their retention times. Rutin at 100 ppm had a retention time of 6.813 min and a peak area of 39.1909 mAU·min, and isoquercetin (quercetin-3-O-glucoside) had a retention time of 7.033 min and a peak area of 48.4411 mAU·min. However, in the bioconverted samples, the products were presumed to be present at lower concentrations, allowing for clearer separation of the peaks. While the bioconversion of quercetin to isoquercetin by *Bacillus* species has been previously reported, our study extends this knowledge by revealing an additional conversion step from isoquercetin to rutin [26,27].

**Figure 6:**
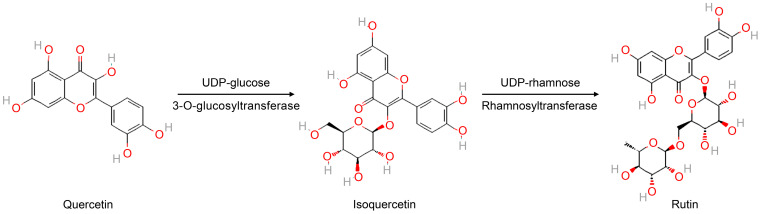
Proposed bioconversion pathway of quercetin to its glycosylated derivatives via enzymatic transformation. Quercetin undergoes glycosylation at the 3-hydroxyl position by glycosyltransferase, forming isoquercetin (quercetin-3-O-glucoside). Subsequent rhamnosylation by rhamnosyltransferase or related enzymes leads to the formation of rutin (quercetin-3-O-rutinoside). These glycosylated products exhibit improved solubility, stability, and bioavailability compared to the aglycone form. Chemical structures: quercetin (CID: 5280343), isoquercetin (CID: 5280804), and rutin (CID: 5280805). Structures were retrieved from the PubChem database (https://pubchem.ncbi.nlm.nih.gov, accessed on 12 April 2025).

The bioconversion of quercetin to its glycosylated derivatives, such as isoquercetin and rutin, is an important metabolic transformation that enhances the solubility, stability, and bioavailability of flavonoids. As shown in the schematic (Figure 6), quercetin can be enzymatically converted to isoquercetin via the addition of a glucose moiety at the 3-OH position through the action of a glycosyltransferase [28,29,30]. Further glycosylation, involving rhamnose attachment to the glucose, leads to the formation of rutin. This stepwise glycosylation pathway is representative of microbial bioconversion processes, in which specific enzymes catalyze the transfer of sugar residues to flavonoid backbones. In this study, we observed a similar conversion pattern using the isolated bacterial strain, suggesting the presence of functional glycosylation enzymes that facilitate the transformation of quercetin to isoquercetin and rutin under in vitro fermentation conditions.

## 4. Conclusions

In this study, a flavonoid-converting bacterium, strain 3P-1, was isolated from buckwheat farmland soil. This strain, identified as closely related to *Bacillus licheniformis* (98.9% similarity), exhibited the ability to bioconvert quercetin into isoquercetin under in vitro fermentation conditions. The conversion was confirmed through HPLC and mass spectrometry, and isoquercetin was identified as the major bioconversion product based on its molecular weight and biosynthetic plausibility. Isoquercetin, a glycosylated derivative of quercetin, is known to have improved physicochemical properties, including enhanced water solubility and intestinal absorption. The ability of strain 3P-1 to produce this compound highlights its potential utility in functional food or nutraceutical applications. However, limitations such as incomplete structural confirmation of the product and the need for precise taxonomic characterization of the strain remain. Further research involving enzyme characterization, metabolite purification, and genome analysis will be necessary to validate and optimize this microbial bioconversion system for industrial use.

## Figures and Tables

**Table 1:** Putative bioconversion production material.

Retention Time	Putative Molecular Weight (*m*/*z*)	Putative Chemical Compositions	Putative Molecular Weight Based on Ion Formula
5.6	437.275	C_21_H_41_O_9_	437.2756
	455.2130	C_17_H_23_N_14_O_2_	455.2134
		C_16_H_27_N_10_O_6_	455.2121
		C_19_H_35_O_12_	455.2134
	457.2440	C_23_H_37_O_9_	457.2443
	463.0872	C_21_H_19_O_12_	463.0882
		C_18_H_11_N_10_O_6_	463.0869
		C_19_H_7_N_14_O_2_	463.0882
	586.2866	C_28_H_44_NO_12_	586.2869
		C_25_H_36_N_11_O_6_	586.2856
		C_26_H_32_N_15_O_2_	586.2869
	643.3115	C_32_H_39_N_10_O_5_	643.3110
		C_33_H_35_N_14_O	643.3124
	666.3451	C_27_H_40_N_17_O_4_	666.3455
		C_26_H_44_N_13_O_8_	666.3441
5.8	463.0872	C_21_H_19_O_12_	463.0882
		C_18_H_11_N_10_O_6_	463.0869
		C_19_H_7_N_14_O_2_	463.0882
	563.1039	C_22_H_15_N_10_O_9_	563.1029
		C_23_H_11_N_14_O_5_	563.1042
		C_25_H_23_O_15_	563.1042

## Data Availability

The original contributions presented in this study are included in the article. Further inquiries can be directed to the corresponding author.

## Abbreviations

-OH	Hydroxyl
HPLC	High-performance liquid chromatography
LC/Q-TOF	Liquid chromatography/quantitative time-of-flight mass spectrometry
MS	Mass spectrometry
PBS	Phosphate-buffered saline
PCR	Polymerase chain reaction
TOF/MS	Time-of-flight mass spectrometry
